# Role of an Automated Deep Learning Algorithm for Reliable Screening of Abnormality in Chest Radiographs: A Prospective Multicenter Quality Improvement Study

**DOI:** 10.3390/diagnostics12112724

**Published:** 2022-11-07

**Authors:** Arunkumar Govindarajan, Aarthi Govindarajan, Swetha Tanamala, Subhankar Chattoraj, Bhargava Reddy, Rohitashva Agrawal, Divya Iyer, Anumeha Srivastava, Pradeep Kumar, Preetham Putha

**Affiliations:** 1 Aarthi Scans & Labs, Chennai 600026, India; 2 Qure.ai, Mumbai 400063, India

**Keywords:** chest X-rays, deep learning, qXR, computer aided diagnostic, neural network, multicenter prospective study

## Abstract

In medical practice, chest X-rays are the most ubiquitous diagnostic imaging tests. However, the current workload in extensive health care facilities and lack of well-trained radiologists is a significant challenge in the patient care pathway. Therefore, an accurate, reliable, and fast computer-aided diagnosis (CAD) system capable of detecting abnormalities in chest X-rays is crucial in improving the radiological workflow. In this prospective multicenter quality-improvement study, we have evaluated whether artificial intelligence (AI) can be used as a chest X-ray screening tool in real clinical settings. **Methods:** A team of radiologists used the AI-based chest X-ray screening tool (qXR) as a part of their daily reporting routine to report consecutive chest X-rays for this prospective multicentre study. This study took place in a large radiology network in India between June 2021 and March 2022. **Results:** A total of 65,604 chest X-rays were processed during the study period. The overall performance of AI achieved in detecting normal and abnormal chest X-rays was good. The high negatively predicted value (NPV) of 98.9% was achieved. The AI performance in terms of area under the curve (AUC), NPV for the corresponding subabnormalities obtained were blunted CP angle (0.97, 99.5%), hilar dysmorphism (0.86, 99.9%), cardiomegaly (0.96, 99.7%), reticulonodular pattern (0.91, 99.9%), rib fracture (0.98, 99.9%), scoliosis (0.98, 99.9%), atelectasis (0.96, 99.9%), calcification (0.96, 99.7%), consolidation (0.95, 99.6%), emphysema (0.96, 99.9%), fibrosis (0.95, 99.7%), nodule (0.91, 99.8%), opacity (0.92, 99.2%), pleural effusion (0.97, 99.7%), and pneumothorax (0.99, 99.9%). Additionally, the turnaround time (TAT) decreased by about 40.63% from pre-qXR period to post-qXR period. **Conclusions:** The AI-based chest X-ray solution (qXR) screened chest X-rays and assisted in ruling out normal patients with high confidence, thus allowing the radiologists to focus more on assessing pathology on abnormal chest X-rays and treatment pathways.

## 1. Introduction

Chest radiography (chest X-ray or CXR) is the most common, economic, and low-radiation imaging modality, with a yearly estimate of over 2 billion imagings performed worldwide [1,2]. CXR serves as a primary and crucial screening modality in the diagnosis and management of cardiothoracic and pulmonary abnormalities, such as pneumothorax, pleural effusion, atelectasis, cardiac hypertrophy, hyperinflation, and consolidation, etc. [3]. CXR plays an important role in the diagnostic domain of medical practice. However, correctly identifying the abnormalities on CXR is often a significant challenge due to the limited number of well-trained radiologists and the heavy workload in extensive healthcare facilities [4]. Additionally, interpretations of CXR are subjective in nature. The complexity of accurate CXR interpretation is largely increased by the presence of overlapping tissue structures. For example, when the lesion overlaps the ribs or large pulmonary blood arteries, or when there is minimal contrast between the lesion and the surrounding tissue, clear identification of the condition might be very difficult [5]. Sometimes, well-trained radiologists find it challenging to differentiate between the lesions or correctly identify very obscure pulmonary nodules [6]. Therefore, the chances of misdiagnosing lung abnormalities in CXR are high. To overcome these challenges, an automated system that can identify abnormalities on CXR with high accuracy and reliability is required. The automated CXR screening tool will aid in decreasing the workload and reducing the potentially missed findings in CXR. Recently, WHO also recommended using AI-based triaging as a potential solution in diagnostic pathways for tuberculosis [7]. The recent advancement of deep learning in various fields, such as computer vision, speech recognition, natural language processing, and bioinformatics, has been promising [8]. However, in contrast to deep learning, which is based on an automated feature learning process, machine learning relies on handcrafted, feature-based engineering that becomes vulnerable when applied to distinct patient groups and varying image qualities. Deep convolutional neural networks (CNNs) have proven to be more powerful in learning hierarchically rich deep features from labelled images. CNN performs a series of convolutional and non-linear operations by optimizing certain loss functions and mapping to the ground-truth labels to bridge the gap between high- and low-level visual feature representations [9].

In recent years, automated CXR screening using CAD systems based on CNNs for the detection of thoracic abnormalities has been an active area of research [10,11]. The CNN-based CAD system is generally trained on a large-scale database of labelled images. This helps in improving the learning of underlying complex semantic features for superior generalization in disease detection. Prior work based on large-scale open-source CXR datasets such as “NIH ChestX-ray 14”, “ChestXpert”, and “MIMIC-CXR” has been conducted by using the CAD system to detect different lung abnormalities i.e., pleural effusion, cardiomegaly, tuberculosis, lung cancer, pulmonary edema, pneumothorax, and pneumonia [12,13,14,15,16,17]. However, in most earlier studies, triaging AI for detecting chest abnormalities from open-source CXR was designed as a retrospective study. In this, ground truth was established based on the already available labelled images or based on the opinion of the minimum number of radiologists. Generally, in such a retrospective setting, the inclusion of CXR is based on a particular disease of interest and a specified number of patients with and without the condition. As a result, the true disease status of the patient at the time of study is generally not known. However, in a prospective design, a patient sample is better characterized in terms of clinical characteristics, standardized techniques for carrying out and interpreting the test(s) and establishing the gold standard process [18]. Moreover, in terms of hierarchy in the importance of evidence, prospective study designs are ranked higher than retrospective study designs as the level of evidence is of higher relevance [19].

The commercially available deep learning-based AI algorithm qXR (Qure.ai Technologies, Mumbai, India) [20] has been used in multiple studies previously in CXR screening for diagnosis of tuberculosis [21], missed or mislabelled findings [22], severity assessment of pneumonia with the need for mechanical ventilation [23], and identification of malignant nodules [24]. We conducted a prospective multicentre quality-improvement study. The study aimed to evaluate the quality of CXR analysis software (qXR) for predicting normal and abnormal CXR in routine screening. The study compared the performance of qXR with the radiologist in triaging and interpreting CXR in a high-CXR volume facility. The qXR also provided an AI-generated report detailing the abnormalities and localization of the abnormality present on the CXR, which assisted the frontline radiologists in patient diagnosis and reduced interreader variability across the readers. To the best of our knowledge, there is no prior publication on triaging AI in routine clinical practice for normal and abnormal reporting from CXR in a multi-centers prospective design.

## 2. Materials and Methods

**Figure 1:**
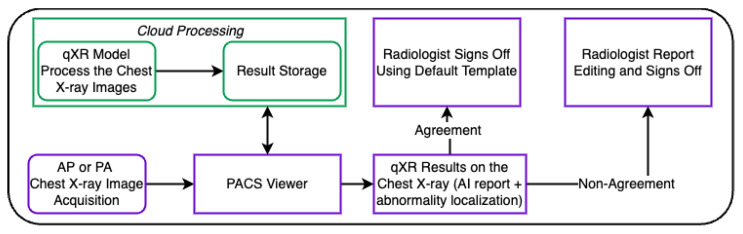
Flow diagram illustrating qXR assisted reporting process followed during this study across different study sites.

The CXR and their corresponding radiological reports were obtained prospectively from the 35 centres of large radiology network in India from June 2021 to March 2022. A total of 10 radiologists with varying levels of experience read the CXR. The inclusion criteria of the study included CXR of patients with posterior–anterior (PA) or anterior–posterior (AP) views, image resolution of 1440×1440 pixels, and de-identified DICOMs(.dcm) format and more than six years of age. Patients were included in the final analysis if the CXR meet the inclusion criteria. The exclusion criteria included lateral view of the chest, incomplete view of lung, incomplete metadata, non-chest radiographs, and CXR with postoperative defects and excessive motion artifacts. Technical integration was tested prior to the start of the study to establish a connection between the picture archiving and communication system (PACS) and CAD software. This ensured that CXRs are auto-pushed from PACS. qXR was set up and installed on the radiologists’ workstations after a successful testing phase. qXR is compatible and can integrate with any PACS system to maintain the data integrity without the substantial change in existing workflow. In this study, qXR classified CXR into three category settings—normal, abnormal, and to be reviewed (TBR)—as the aim was to filter the normal scans with high confidence. In Figure 1, the process followed at different study sites is given.

**Figure 2:**
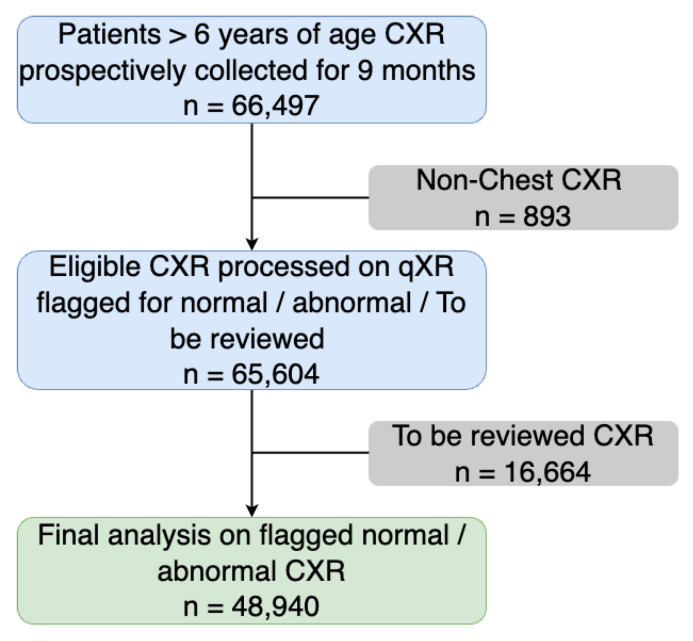
A consort diagram determining the exclusion criteria and establishing the final study population.

The qXR processed all 65,604 CXR and categorised them as normal, abnormal, or TBR by the radiologist. TBR are those cases wherein qXR flagged CXR for escalated radiologist’s review. A total of 16,664 CXR were categorized as TBR. A total of 48,940 CXR were either classified as normal (qXR predicted no significant abnormality) and abnormal when qXR predicted significant abnormality. These cases were used for final analysis to determine the performance of the qXR. In Figure 2 a detailed semantic diagram on the final study population is given. The natural language processing (NLP) was implemented to parse the radiological report. The normal CXR comprised of no detectable abnormalities in the airways, lung fields, cardiac region, mediastinum, pleura, and visible thoracic skeleton and diaphragm. CXR which were not normal were categorized as abnormal. The major abnormalities which were clubbed in the abnormal category included blunted costo phrenic angle, hilar dysmorphism, cardiomegaly, reticulonodular pattern, rib fracture, scoliosis, atelectasis, calcification, consolidation, emphysema, fibrosis, nodule, opacity, pleural effusion, and pneumothorax. All the radiologists reading the CXR across multiple radiological centres had access to automated qXR diagnostic report on reader’s computers. The radiologist’s reports were finally used as a reference criterion to compare qXR performance in classifying normal and abnormal CXR by using standard evaluation metrics.

### Statistical Analysis

Statistical analyses were performed by using Python version 3.9.7, Programming Language. The performance of qXR in categorizing normal and abnormal CXR was evaluated by using standard evaluation metrics, i.e., sensitivity, specificity, negative predictive value (NPV), and area under the ROC curve (AUC). These 95% confidence intervals for all performance evaluating metrics have been estimated by using the Wilson score method [25,26] without continuity correction.

## 3. Results

The final study population resulted in 65,604 CXR from 35 study centers in six different states of India after excluding 893 CXR as per exclusion criteria. Among the 16,664 TBR cases, 15,019 (90.13%) were marked as normal and 1645 (9.87%) were abnormal by the radiologists. To reduce potential bias in assessing the performance of the qXR, the final analysis is based upon 48,940 CXR after excluding all the TBR cases. The median age of the study population was 42 years. The mean, standard deviation, and interquartile range of patient age were 43.36 and 16.32, and 24. There were 61.7% male and 38.3% female patients included in the study. The automated qXR successfully sent all the processed CXR results to the PACS for easy visualization of outputs for the radiologists. The qXR achieved an overall mean sensitivity and specificity of 87.9% and 82.9%, respectively, in predicting normal and abnormal CXR. The high mean NPV of 98.9% suggests the efficacy of qXR in ruling out normal patients, thus allowing the radiologists to focus more on assessing pathology on abnormal CXR and treatment pathways. In Table 1, the detailed performance of qXR in categorizing normal and abnormal CXR is given.

**Figure 3:**
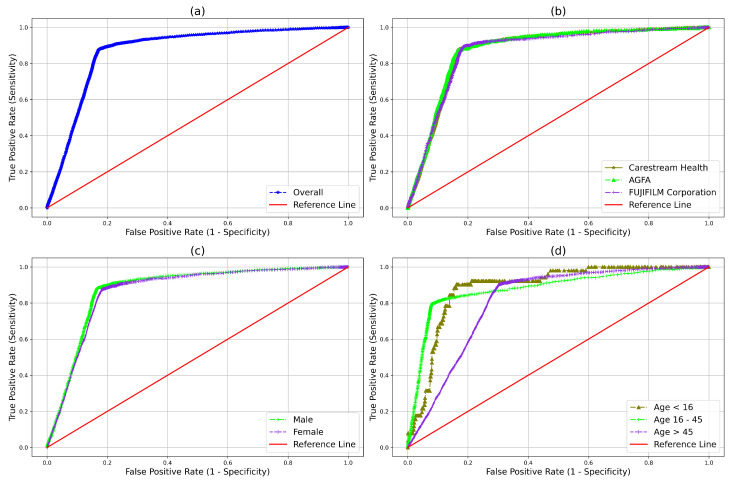
ROC curve. (**a**) Overall qXR performance. (**b**) Stratified across three different manufacturer subgroups. (**c**) Stratified across gender. (**d**) Stratified across 3 different age subgroups.

The robustness of the qXR is further analyzed by stratified performance comparison in terms of NPV, sensitivity, specificity, and AUC across three different manufacturers, age in three different subgroups (16 years and less, 16 to 45 years, and more than 45 years of age), and gender is given in Table 2. It is observed from Table 2 that the NPV for different manufacturers obtained by qXR in categorizing normal or abnormal is 98.8%, 98.7%, and 99.1%. From three different age groups, the values are 99.5%, 99.3% and 98.1%. From gender, the values are male, 99% and female, 98.7%. The stratified analysis conveys the high-performance stability of qXR across subgroups and usability in ruling out normal in routine CXR screening. In Figure 3 the ROC curves for qXR overall performance, stratified by manufacturer and patient’s demographic is also given.

**Figure 4:**
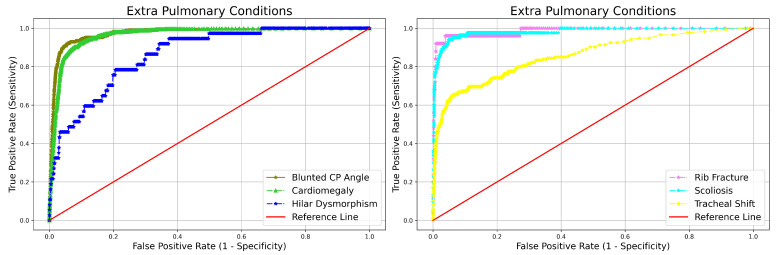
ROC curve qXR performance in identifying subabnormality from abnormal CXR and categorizing into extra pulmonary.

**Figure 5:**
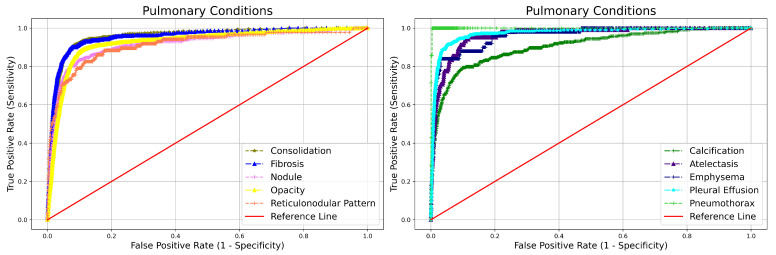
ROC curve qXR performance in identifying subabnormality from abnormal CXR and categorizing into pulmonary conditions.

The CXR categorized as abnormal are further classified into two main categories and 15 major subabnormalities. The remarkable performance of qXR in terms of NPV for different abnormalities i.e., blunted CP angle (99.5%), hilar dysmorphism (99.9%), cardiomegaly (99.7%), reticulonodular pattern (99.9%), rib fracture (99.9%), scoliosis (99.9%), atelectasis (99.9%), calcification (99.7%), consolidation (99.6%), emphysema (99.9%), fibrosis (99.7%), nodule (99.8%), opacity (99.2%), pleural effusion (99.7%), and pneumothorax (99.9%) can be observed from Table 3. Furthermore, the capability of qXR in distinguishing the presence or absence of specific abnormalities like cardiomegaly, nodule, and pneumothorax in terms of AUC is 0.965, 0.915, and 0.999 can be observed from Table 3. The ROC curve qXR performance in identifying subabnormality from abnormal CXR is given in Figure 4 and Figure 5.

**Figure 6:**
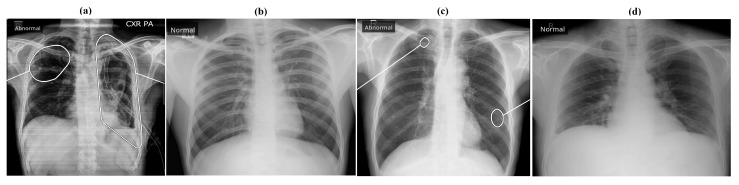
The CXR classified by qXR as (**a**) true positive (TP), (**b**) true negative (TN), (**c**) false positive (FP), and (**d**) false negative (FN). (**a**) Predicted as abnormal with impression of cavity, pleural effusion, pneumothorax, opacity, consolidation and fibrosis in the left lung while cavity and opacity were also predicted in the right lung. (**b**) Predicted as normal. (**c**) Predicted as abnormal with pleural effusion, pneumothorax and opacity in the left lung and right lung. (**d**) Predicted as normal.

There were total of 410 CXRs which qXR predicted as normal but were marked as abnormal by the radiologist. Representative CXR processed by the qXR, predicted as normal and abnormal along with abnormality localization is represented in Figure 6.

### Turnaround-Time Analysis

The baseline i.e., pre-qXR data was procured from the radiological scan centres during a three-month period. After the qXR was deployed i.e., post-qXR, we obtained the data randomly from the 48,940 CXR that was considered in final analyis for comparing difference in radiology turnaround time (TAT) between the two periods. TAT as the difference between time at which the final CXR report was reported by the radiologist and the time at which the CXR was assigned to the radiologist. The unpaired *t*-test is used to compute the statisctial signficance of the change in TAT for pre-qXR and post-qXR [27].

There were a total of 18,496 CXRs (9248 (50%) pre-qXR, and 9248 (50%) post-qXR deployment) considered for this analysis. The mean TAT decreased by about 40.63% (83.028 min in the pre-qXR period and 50.287 min in the post-qXR period). This reduction was significant based on the unpaired *t*-test (*p*-value <0.0000001). The summary statistics of TAT between the groups are given in the Table 4.

## 4. Discussion

In this prospective multicenter quality-improvement study, we have analyzed CXR with the help of a commercially available deep learning-based AI algorithm (qXR). The purpose was to identify normal and abnormal findings and to demonstrate the efficacy of triaging AI in routine CXR screening. The high NPV (98.9%) in categorizing normal and abnormal CXR demonstrates the utility of qXR as a screening tool in high-volume facilities. In a prior study, deep learning algorithms were used for interpreting 420 CXRs interpretation from a publicly available database. The reported sensitivity for atelectasis, cardiomegaly, edema, and pleural effusion was 0.750, 0.617, 0.712, and 0.806, respectively [28]. In another study using three AI algorithms for CXR interpretations, 13 abnormalities were reported with a mean AUC for DenseNet121, InceptionResNetV2, ResNet152V2 was 0.793, 0.801, and 0.751, respectively [29]. Prior studies have reported the use of AI as a standalone or a second reader [30] with comparable performance, but validation of such tools in real clinical settings is still missing and represents an active area of research.

Most AI models suffer from poor generalization due to target domain divergence. Previously, the qXR has been evaluated retrospectively in more than 2.3 million CXR, where it categorized CXR into normal and different abnormalities [20]. To determine whether an AI algorithm can be utilized in real clinical settings, a validation of its performance in the real world is required. The added advantage of our study from previously published literature [20,31,32,33] is that the AI (qXR) has been evaluated prospectively in clinical settings. The algorithm showed high efficacy in classifying CXR into clinically relevant abnormalities, demonstrating its usability in real-time routine CXR screening in large clinical facilities. The overall sensitivity and specificity obtained is 87.9% and 82.9%, respectively. In general clinical practice, CXR serves as a preliminary screening examination for different thoracic and pulmonary abnormalities, where the sensitivity metric is more important, especially in high volume and low-resource facilities. Additionally, the minimum number of false negatives of 0.83% demonstrates its potential as a computer-aided diagnosis tool in emergency departments to improve CXR interpretations. Among the different types of abnormalities, the NPV obtained was 99.9% in almost all abnormalities. Additionally, the high AUC score obtained for different abnormalities shows the capability of the qXR in separating and categorizing different abnormalities. However, among all the abnormalities, the hilar dysmorphism, and reticulonodular pattern achieved a least AUC score of 0.864, 0.913 respectively. This may be attributed to the limited number of true positive samples, i.e., 37, for hilar dysmorphism, and 86 for reticulonodular pattern. In the subgroup analysis, the qXR showed consistent performance in terms of NPV for different subgroups in manufacturer, age, and sex, demonstrating the AI algorithm’s robustness.

In the conventional clinical approach, there is minimal use of technology. However, there are many advantages to using AI in radiology departments. With the usage of AI, the delivery of healthcare services to the patient can become more efficient and timely. AI can reduce wait times for patients to get the final report, especially for normal cases, as we have demonstrated in this study. In a conventional system, the normal and abnormal CXR are in the same worklist, and there is no way to segregate/triage normal CXR without opening the CXR. AI as a secondary reader assists in reducing errors in the reports and missed diagnoses [22]. Beyond reduction in reporting time and improvement in report quality, the use of AI will lead to more appropriate treatments for the patients in a timely manner. This is also expected to be reflected in better clinical outcomes downstream in care management. Adoption of AI in radiology can improve quality metrics and volume of reports significantly over time. This will benefit the radiologists, different department levels of the healthcare facilities, and also the end-to-end patient care.

Although there are many advantages to using technology, some costs/efforts are associated with introducing any innovation. Every innovation has upfront costs, and the benefits are realized over time. Therefore, some physicians and departments might want to wait before they choose to spend to adopt this innovation [34]. Overall, the advantages are much longer than of inconveniences; this study also demonstrates robust evidence to make a case for quicker adoption of AI in radiology. AI not only adds to the efficiency for the clinicians, but a faster adoption is ethical so that the benefits of innovation are passed on to the patients who can benefit from precise, timely, and better care.

There were certain limitations in our study. First, our study was based on a single demographic and racial category; thus, whether the performance of the qXR is reproducible in another demographic and racial category needs to be validated. Secondly, the number of true positives samples was only 6.9% of the final study population. A larger number of true positives would provide more exhaustive testing and further analysis of the qXR. Finally, we want to improve the qXR algorithm and fine tune the threshold to increase the normal CXR prediction, which is truly normal and thus decrease the radiologist’s workload further. In the future, it would be beneficial for us to conduct a study with a larger sample and more findings. Another helpful step would be further developing qXR at larger hospitals in emergency settings to measure its performance and compare the TAT in reducing the radiologist’s workload.

## 5. Conclusions

This study has prospectively demonstrated that using AI as an assistance tool can be beneficial in high-workload healthcare facilities. In this multicenter prospective study, the high NPV obtained for overall and multiple abnormalities indicates the use of AI in finding and localizing the abnormalities on CXR. The remarkable AUC score obtained for different clinically relevant abnormalities shows the capability of the AI tool in categorizing the CXR with multiple abnormalities. The triaging of AI in routine CXR screening becomes more critical for developing and underdeveloped countries with a shortage of skilled radiologists. AI tools with high NPV like qXR can be utilized for screening purposes to screen out normal patients, thus allowing clinicians to focus more on patients with abnormalities and their treatment pathways. Additionally, the AI as a second reader enables radiologists to decide rapidly with higher confidence and thus reduce the interrater variability and workload in high CXR volume facilities.

## Figures and Tables

**Table 1:** Performance of qXR in categorizing normal or abnormal CXR using radiologist as reference standard. negative predictive value (NPV); confidence interval (CI).

Evaluation Metrics	Point Estimate (95% CI)
Sensitivity	0.879 (0.867–0.889)
Specificity	0.829 (0.825–0.832)
NPV	0.989 (0.981–0.990)
AUC	0.871 (0.866–0.877)

**Table 2:** Performance of qXR in categorizing normal or abnormal CXR stratified by patient’s demographic and manufacturer using radiologist as reference standard. Negative predictive value (NPV); Area under the curve (AUC).

Attributes		NPV	Sensitivity	Specificity	AUC
Manufacturer	Carestream Health	**0.988** (0.987–0.990)	0.878(0.862–0.892)	0.831 (0.826–0.836)	0.872 (0.862–0.883)
	AGFA	0.987 (0.985–0.989)	0.865 (0.840–0.887)	**0.836** (0.829–0.843)	**0.878** (0.862–0.893)
	Fujifilm	0.991 (0.989–0.993)	**0.894** (0.870–0.914)	0.817 (0.810–0.824)	0.868 (0.851–0.884)
Age (years)	16 and less	**0.995**(0.988–0.997)	0.901 (0.790–0.957)	0.819 (0.797–0.840)	**0.886** (0.826–0.946)
	16–45	0.993 (0.992–0.994)	0.792 (0.763–0.819)	**0.922** (0.919–0.925)	0.878 (0.862–0.893)
	45 and above	0.981 (0.978–0.983)	**0.905** (0.893–0.916)	0.694 (0.688–0.701)	0.809 (0.798–0.819)
Gender	Male	**0.990** (0.989–0.991)	**0.884** (0.869–0.897)	**0.833** (0.829–0.838)	**0.875** (0.865–0.885)
	Female	0.987 (0.985–0.989)	0.871 (0.853–0.888)	0.821 (0.815–0.826)	0.866 (0.854–0.878)

**BOLD** denotes best performance in the stratified category for the different metrics.

**Table 3:** Performance of qXR in categorizing abnormal CXR into subabnormalities by using radiologist as reference standard. Negative predictive value (NPV). Area under the curve (AUC).

	Abonrmality	NPV	Sensitivity	Specificity	AUC
	Blunted CP angle	0.995 (0.995–0.996)	0.484 (0.435–0.534)	0.990 (0.989–0.991)	0.973 (0.709–0.766)
	Hilar Dysmorphism	0.999 (0.999–0.999)	0.216 (0.113–0.371)	0.992 (0.991–0.993)	0.864 (0.789–0.939)
Extra Pulmonary	Cardiomegaly	0.997 (0.997–0.997)	0.804 (0.770–0.835)	0.962 (0.960–0.964)	0.965 (0.955–0.975)
	Reticulonodular Pattern	0.999 (0.998–0.999)	0.511 (0.407–0.614)	0.983 (0.981–0.984)	0.913 (0.872–0.954)
	Rib Fracture	0.999 (0.999–0.999)	0.840 (0.653–0.935)	0.991 (0.991–0.992)	0.984 (0.951–1.000)
	Scoliosis	0.999 (0.999–0.999)	0.698 (0.593–0.786)	0.995 (0.995–0.996)	0.981 (0.961–1.000)
	Atelectasis	0.999 (0.998–0.999)	0.607 (0.5108–0.697)	0.982 (0.980–0.983)	0.962 (0.936–0.987)
	Calcification	0.997 (0.997–0.997)	0.804 (0.770–0.835)	0.962(0.960–0.964)	0.965 (0.955–0.975)
	Consolidation	0.996 (0.995–0.996)	0.702 (0.663–0.737)	0.967 (0.966–0.969)	0.956 (0.944–0.967)
	Emphysema	0.999 (0.999–0.999)	0.580 (0.442–0.706)	0.988 (0.987–0.989)	0.960 (0.922–0.998)
Pulmonary	Fibrosis	0.997 (0.996–0.997)	0.650 (0.598–0.698)	0.977 (0.976–0.978)	0.955 (0.940–0.970)
	Nodule	0.998 (0.997–0.998)	0.719 (0.667–0.766)	0.955 (0.953–0.956)	0.915 (0.894–0.936)
	Opacity	0.992 (0.991–0.993)	0.828 (0.811–0.844)	0.921 (0.919–0.924)	0.925 (0.917–0.933)
	Pleural Effusion	0.997 (0.996–0.997)	0.667 (0.619–0.712)	0.986 (0.985–0.987)	0.972 (0.961–0.984)
	Pneumothorax	0.999 (0.999–0.999)	0.857 (0.486–0.974)	0.998 (0.998–0.998)	0.999 (0.983–1.000)

**Table 4:** Summary statistics of TAT analysis between pre-qXR and post-qXR.

Attributes	Pre-qXR (minutes)	Post-qXR (minutes)
Minimum	11.547	**6.249**
Mean	83.028	**50.287**
Maximum	24,918.617	**14,290.85**

**BOLD** denotes least time in minutes.

## Data Availability

Not applicable.
